# Physiological evaluation of the filamentous fungus *Trichoderma reesei *in production processes by marker gene expression analysis

**DOI:** 10.1186/1472-6750-7-28

**Published:** 2007-05-30

**Authors:** Jari J Rautio, Michael Bailey, Teemu Kivioja, Hans Söderlund, Merja Penttilä, Markku Saloheimo

**Affiliations:** 1 VTT Technical Research Centre of Finland, Tietotie 2, Espoo, PO Box 1000, 02044 VTT-Espoo, Finland

## Abstract

**Background:**

Biologically relevant molecular markers can be used in evaluation of the physiological state of an organism in biotechnical processes. We monitored at high frequency the expression of 34 marker genes in batch, fed-batch and continuous cultures of the filamentous fungus *Trichoderma reesei *by the transcriptional analysis method TRAC (TRanscript analysis with the aid of Affinity Capture). Expression of specific genes was normalised either with respect to biomass or to overall polyA RNA concentration. Expressional variation of the genes involved in various process relevant cellular functions, such as protein production, growth and stress responses, was related to process parameters such as specific growth and production rates and substrate and dissolved oxygen concentrations.

**Results:**

Gene expression of secreted cellulases and recombinant *Melanocarpus albomyces *laccase predicted the trends in the corresponding extracellular enzyme production rates and was highest in a narrow "physiological window" in the specific growth rate (μ) range of 0.03 – 0.05 h^-1^. Expression of ribosomal protein mRNAs was consistent with the changes in μ. Nine starvation-related genes were found as potential markers for detection of insufficient substrate feed for maintaining optimal protein production. For two genes induced in anaerobic conditions, increasing transcript levels were measured as dissolved oxygen decreased.

**Conclusion:**

The data obtained by TRAC supported the usefulness of focused and intensive transcriptional analysis in monitoring of biotechnical processes providing thus tools for process optimisation purposes.

## Background

Microorganisms used in various types of biotechnical processes encounter constantly changing environmental conditions, to which they adapt by changing their cellular physiology. The performance of the microorganism has a major impact on the performance of the process and as a consequence, bioprocess monitoring and control strategies based on the physiological status of the culture have become more popular [1,2]. In bioprocesses the physiological status of the culture is generally measured indirectly by analysis of extracellular variables such as enzyme activities, pH and exhaust gas concentrations. Recent developments in analytical methods allow direct evaluation of cellular physiology by analysis of intracellular variables such as proteins, RNAs or metabolites, either in focused or systems-wide manner [1,3].

By combination of transcriptomic, proteomic and/or metabolic profiles with advanced bioinformatic analysis it is possible to predict from the massive amount of data biologically meaningful analytes, *i.e*. biomarkers which can predict certain physiological events. In biotechnical processes such biomarkers have been used *e.g*. for detection of osmotic and oxidative stress conditions or trace element deficiencies in yeasts [2,4] and bacteria [3]. Simple and robust assays are required in order to use these biomarkers in monitoring or control of bioprocesses. Transcriptional analysis of selected marker genes provides one potential way for robust monitoring of physiological events, since adaptation to changes in the environment takes place rapidly at the level of gene expression [5,6]. In addition it is possible to measure multiple targets in a single assay with RNA which is more difficult to achieve with enzymes or metabolites.

Few microarray studies have been performed specifically to identify gene markers to be used in monitoring of industrial fermentations [4,7]. Conventional Northern blot analysis is still abundantly used for the actual monitoring of the selected markers in process conditions [2,4,7]. New tools for more robust RNA analysis are emerging, such as quantitative RT-PCR [8], and combination of sandwich hybridisation with fluorescent [9] or electric detection [10,11]. Depending on the organism and length of the process these methods may be feasible for at-line mRNA expression monitoring, but would generally be applicable for only a limited number of targets and/or samples.

We have recently developed a method for transcriptional analysis method called TRAC (Transcript analysis with aid of affinity capture) [12,13]. The TRAC method is suitable for rapid simultaneous analysis of 96 samples with multiplex target detection, allowing high-throughput, focused expression analysis [12] or alternatively it can be performed with amplifiable DNA probes and PCR [13] to reach high sensitivity. TRAC has been applied for gene expression analysis of microbial cultures [12], multiplex quantification of bacterial populations [14,15] and to monitor gene expression stability in continuous cultures [16]. In this study we have used TRAC for expression monitoring of more than 30 marker genes in different types of protein production processes performed with the industrially important filamentous fungus *Trichoderma reesei*. Expression levels of the marker genes, which are involved in different process relevant pathways, were compared with extracellular process parameters from the cultures, such as specific growth rates, production rates of secreted enzymes and availability of nutrients and oxygen. Identification of relationships between gene expression fingerprints and process performance would offer possibilities for process optimisation and control.

## Results

### polyA RNA- and biomass-based normalisation of gene expression data

Expression of a set of 34 marker genes, representing various physiological states, was monitored in different phases of two batch and fed-batch cultures and one continuous culture of an *M. albomyces *laccase producing strain of the filamentous fungus *Trichoderma reesei*. Potentially useful molecular marker genes were selected on the basis of their responses in conditions of interest as reported in publicly available transcriptional analysis data for various species of filamentous fungi or *Saccharomyces cerevisiae*. The genes used in this study are listed in Table 1 with references to available data on these genes or corresponding homologues in other organisms.

**Table 1:** *T.reesei *genes used in the TRAC analysis grouped into functional categories.

**Gene name**	**Function**	**Probe Length (nt)**	**Probe sequence 5'-3'**	**Location of the probe in CDS**	**Reference data**
**Carbohydrate degradation/oxidation**					
*bgl2*	β- glucosidase	41	CGTTATAGTACTTGACCCTGAAGTCATCTTCGAGAATCTTC	1143–1183	51
*cbh1*	Cellobiohydrolyase	25	CATTCTGGACATAGTATCGGTTGAT	889–913	19
*egl1*	Endoglucanase	27	CGGACTTTGTACACTTGTAGGTTGTCA	107–133	19
*lac1*	*Melanocarpus albomyces *laccase	35	GACCAACGCATGTCGAAAGTGAACAACAAGTAACC	1952–1986	24
**Stress**					
*bip1*	Protein chaperon	27	AGGGGGTTGACGTCCATGAGAACAATG	1008–1034	58
*gcn4*	Transcription factor	37	TGAAGAAGACGATCGGTACATGGGCTCTGATTCCAAA	347–410	52
*hac1*	Transcription factor	31	AGAGAGTGATGCTGTCCTGGAGAGAGTCGAG	562–592	58
*hsp105*	ER chaperon	37	CGGGCTTATCCTCAGTGTCAACTTGTTGATAGAATAA	1522–1558	37
*hsp30*	Heat shock protein 30	31	GTACTTTGCGTTGTCGGTAGGCTTGTTGCTG	750–780	36
*msn4*	Transcription factor	35	CGAGAGAACTTCTTGCCGCACTCGTTGCACTCAAA	1327–1361	5
*nsf1*	General membrane fusion factor	41	CAACAGGGCATCGTCAATCATGTCTTTTCGATTCGTCATTC	1388–1428	59
*nth1*	Neutral trehalase	43	AACGTAACTGGCATTGACCCATCCAAATCCTTCTTTCGCAACG	2061–2103	30
*pdi1*	Protein disulfide isomerase	33	GGTCAAAGGGGAACTTGAGGTTCTTCTCAATGT	926–958	58
*sod2*	Superoxide dismutase	29	TTGATGACGTCCCAGATGGCGCTGAAGTA	637–665	5
*tps1*	Trehalose-6-P-phosphate synthase	41	AACTTGCGGATGAACTTGGTGATCCACGACTGGACATTCTG	1612–1652	53
*trr1*	Thioredoxin reductase	31	AATGACGAAGAGGGGCTTGTTGCGGAAGATG	444–474	18
*trx2*	Thioredoxin protein	39	CAAACTTGACAAAGTGGACCTTGTCCTTGAACTCTGCGT	230–268	18
**Central carbohydrate metabolism**					
*acs1*	Acetyl-CoA-synthethase	39	TTGTGCTTCTCAATAATGTCCCAGTACCTTGAGAAGTTG	1263–1301	17
*eno1*	Enolase	25	TTACGGAAGTTGGTGCCAGCGTAGA	1277–1301	17
*gpd1*	Glyceraldehyde-3-P-dehydrogenase	29	ACGAAGTTGGGGTTCAGGGAGATACCAGC	895–923	17
**Growth and conidiation**					
*ccg9*	Trehalose synthase	39	AAACTTTGACTTCGAACCCTTCATACGTCGACAGTTGAA	902–940	34
*chs1*	Chitin synthase	37	GAAAGAAGCGATAAAGTAGAGGCCGTAAATGGTAATC	2133–2169	54
*con6*	Conidiation related gene	31	TGCTTAGCGTTTTCCTTTGCTTCCTCCGACA	278–308	35
*rpl16a*	Ribosomal protein L13A, 60S subunit	27	CAACCTTCTTGCGCTCGTAGTAGGCAG	1612–1652	55
*rps16b*	Ribosomal protein S16A, 40S subunit	35	TGACACGGACGCGGATGTCGACGTTGGCGAACTTG	174–208	55
**Proteases**					
*aep1*	Extracellular protease	25	CATGGAGGTGCCGCTAATCGTGTTT	1038–1062	56
*axp1*	Extracellular protease	33	AAGTTGAAGGTGGCATCCTTGATGTTTGCTTTG	933–965	56
*mca1*	Metacaspase, cysteine protease	25	AATACCCTGCGTGGAGTAGATGTAC	861–885	33
*vpa1*	Intracellular aspartic protease	27	GTGATGTCGGGGAGGGAATCACGCTTG	957–983	57
**O_2 _regulated**					
*hem6*	Coproporphyrinogen III oxidase	29	ACTTCTTGAACCGAGGGTAGTACGTCTTG	798–826	39
*hsp70*	Heat shock protein 70	33	TTGGTGATGACAATCTTGTTGGACTTACCAGTG	1545–1577	40
**Transport**					
*ctaA*	ATPase copper transport	41	ACGAGTGATTGTGCCGGTTTTGTCCAAGACGACTTTGGTAA	2309–2349	31
*gap1*	Amino acid transporter	33	TGATACTTCCAGGCATTGCGGAATCGGATGTGG	1335–1367	52

The TRAC expression analysis for the selected genes was performed directly from cell lysates, and the gene expression levels were related to the amount of biomass used in the hybridisation or to overall polyA RNA concentration of the lysate (see Methods). The ratio of marker gene mRNA to total polyA RNA shows the change in the expression of a specific gene relative to overall gene expression, whereas the ratio of marker gene mRNA to biomass shows the changing level of a transcript relative to the overall culture (viable/non-viable).

**Figure 1:**
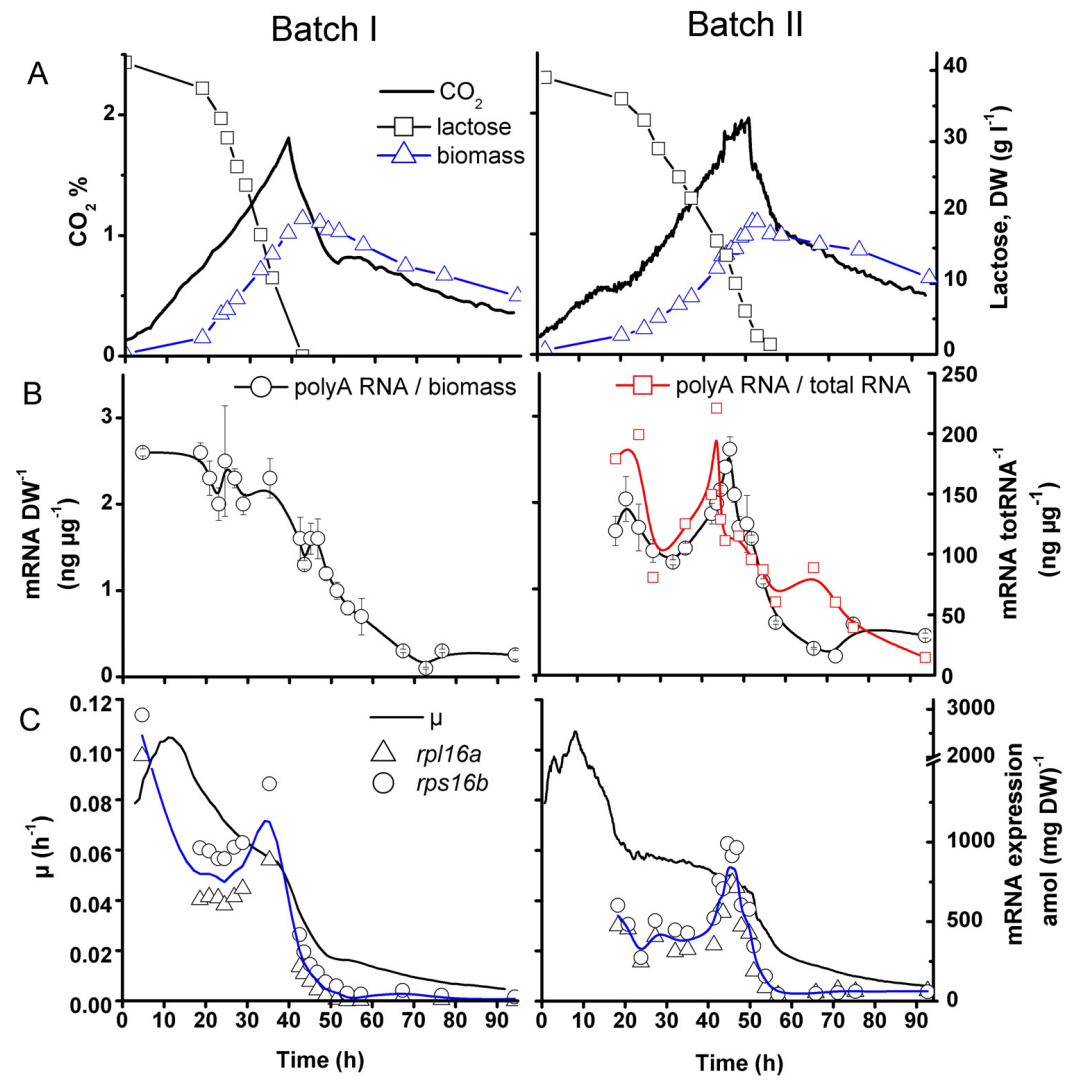
**PolyA RNA and ribosomal protein mRNA levels in twobatch cultures of *T. reesei***. A. Lactose, biomass and CO_2 _concentrations in batch cultures I and II. B. PolyA RNA concentration relative to biomass in cultures I and II and polyA RNA concentration relative to total RNA in culture II. C. Specific growth rate (μ) and expression of two ribosomal protein mRNAs (*rpl16a*, *rps16b*) relative to biomass. Cultures were maintained at 28°C and pH 5.5 – 6.

PolyA RNA concentration in the lysed biomass in different growth phases of the two batch cultures, which differed considerably in growth and substrate consumption rate are presented in Figure 1. In batch I the polyA RNA concentration varied between 2 and 2.5 mg g^-1 ^dry weight (DW) for the first 35 h, while μ was above 0.06 h^-1 ^and lactose concentration above 10 g l^-1^. After 35 h, when the culture conditions had become growth limiting, the polyA RNA content relative to biomass decreased to approx. 0.4 mg g^-1 ^dry weight. However in batch culture II the specific growth rate and uptake of lactose were slower than in batch I after 20 h and the polyA RNA per g biomass decreased at this time already, although the polyA RNA content in the biomass subsequently increased between 30 and 45 h. After 45 h the cultures were similar. To verify that the decrease in the polyA RNA content after 20 h was not a result of experimental error (*e.g*. variation in cell lysis efficiency), total RNA was extracted from the samples of batch culture II and the polyA RNA to total RNA ratio was determined. This confirmed that a decrease in overall gene expression had occurred after 20 h (Figure 1B).

The specific growth rates (μ) were compared to the expression of two ribosomal protein mRNAs (*rpl16a*, *rps16b*) as growth-related marker genes in these batch cultures. The changes in the transcription of these genes relative to biomass were similar to μ (Figure 1C). The relatively low specific growth rate between 20 and 30 h for batch II (25% lower than in batch I), corresponded to a 1.7-fold lower expression of the two ribosomal mRNAs during this period. However, in both fermentations, an approximately 2-fold increase in the ribosomal protein mRNA to biomass ratio was observed 3 to 5 h before the decrease in specific growth rate which occurred after all lactose had been consumed.

### Monitoring carbohydrate metabolism markers

**Figure 2:**
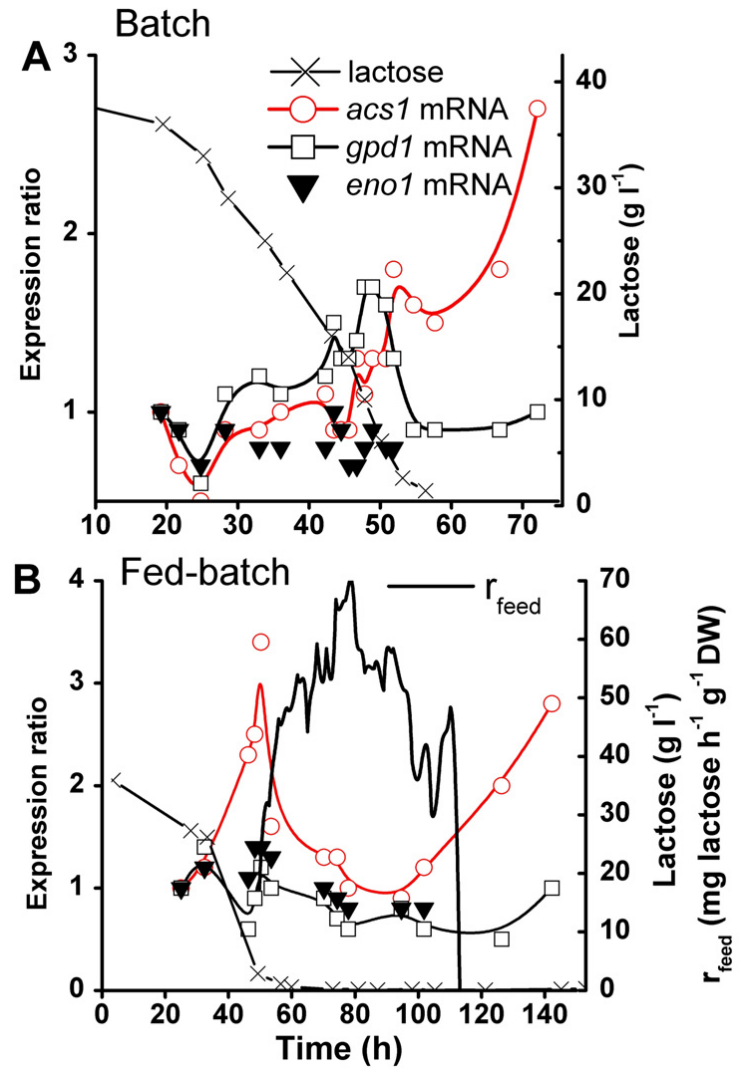
**Expression of carbohydrate metabolism marker genes in a batch culture (A) and a fed-batch culture (B) of *T. reesei***. Lactose concentration, Feed rate of lactose r_feed _(mg lactose h^-1 ^g^-1 ^DW), mRNA expression levels of *acs1*, *gpd1 *and *eno1 *relative to expression level measured in the first sample (19.2 h). Expression levels were normalised using polyA RNA.

A number of genes coding for enzymes involved in central carbohydrate metabolism (glycolysis, TCA cycle) are known to be transcriptionally regulated according to the availability of glucose in *T. reesei *[17]. We measured the relative expression of three of these genes involved in central carbohydrate metabolism in batch and fed-batch fermentations with lactose as carbon source (Figure 2). Lack of glucose has been shown to have an increasing (acetyl-CoA-synthethase, *acs1*), decreasing (enolase, *eno1*) or indifferent effect (glyceraldehyde-3-P-dehydrogenase, *gpd1*) on their expression [17]. Both *gpd1 *and *acs1 *increased from 25 to 45 h in lactose-rich conditions (>10 g l^-1^) in batch cultures (Figure 2A). After the lactose concentration decreased below 10 to 6 g l^-1^, the abundance of *gpd1 *mRNA relative to polyA RNA decreased 30 – 65% compared to its maximal expression around 50 h, whereas *acs1 *mRNA continued to increase at low lactose concentration (below 5 g l^-1^) and during starvation. About 20 h after complete exhaustion of lactose, *acs1 *was expressed at a 2.5 to 3 -fold higher level than in high lactose concentrations. The *eno1 *mRNA to polyA RNA ratio was constant when lactose concentration was above 5 g l^-1^, whereas during starvation *eno1 *mRNA was undetectable.

In the fed-batch fermentations a 2 to 3 -fold increase of *acs1 *mRNA level occurred when lactose concentration decreased from 40 to below 5 g l^-1^, before the start of the lactose feed (Figure 2B) as in the deceleration to stationary phases of the batch cultures. Lactose was fed to the fermentor after 45 h at a rate that maintained the rate of base (NH_4_OH) consumption at around 0.012–0.014 ml l^-1 ^min^1^. As long as the feed rate of lactose was below 30 mg h^-1 ^g^-1 ^DW, *acs1 *mRNA level continued to increase, but it decreased rapidly after the feed rate increased above this value (Figure 2B), even though the residual lactose concentration in the medium remained zero. Termination of the lactose feed caused another increase in *acs1 *expression. *gpd1 *and *eno1 *mRNA levels showed only minor changes during the different phases of the fed-batch cultures, and *eno1 *was again undetectable under starvation conditions.

### Monitoring marker genes responding to oxygen

**Figure 3:**
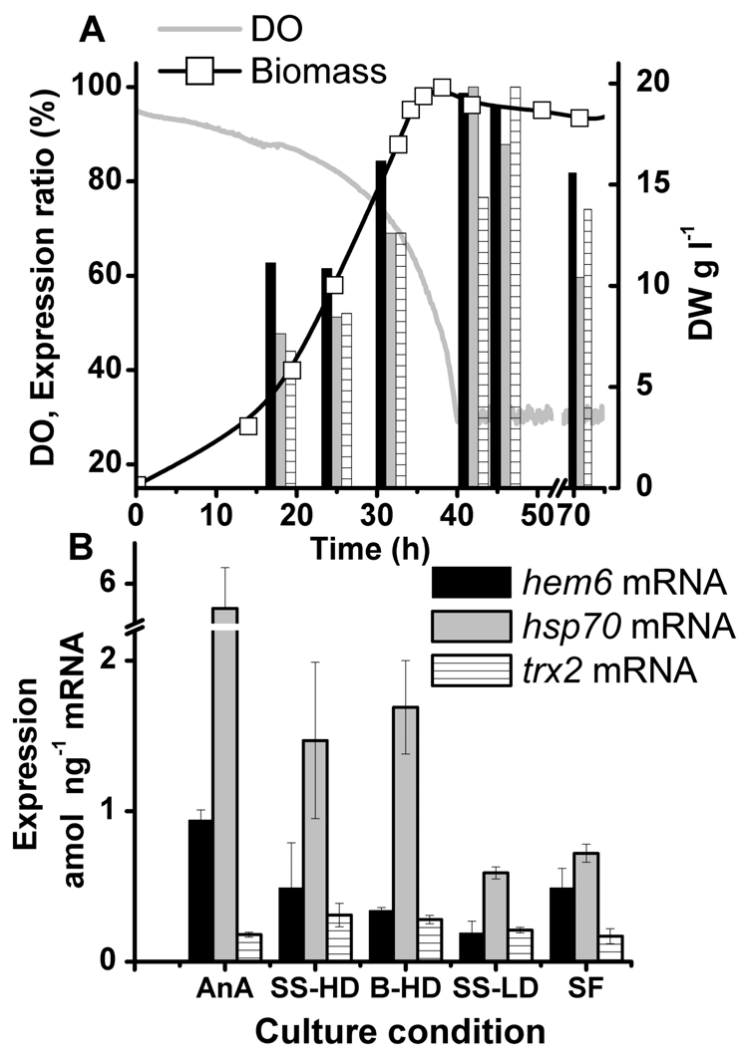
**Expression of oxygen sensitive *hem6, hsp70 *and *trx2 *genes in different cultures of *T. reesei***. Expression of *hem6*, *hsp70 *and *trx2 *genes (A) during batch culture of *T. reesei *compared with DO and biomass concentration and (B) maximal expression levels of *hem6*, *hsp70 *and *trx2 *measured in an anaerobic culture (AnA, DW 4 g l^-1^, pO_2_~0%), steady state with high cell density (SS-HD, DW 16 g l^-1^, pO_2 _~30%) and with low cell density (SS-LD, DW 4 g l^-1^, pO_2 _~80%), in batch cultures with high cell density (B-HD, DW 20 g l^-1^, pO_2 _~30%) and in shake flask precultures (SF, DW ~2 g l^-1^). Error bars show the standard deviation between triplicate cultures.

Expression of the heat shock protein gene *hsp70 *and the heme biosynthesis gene *hem6 *has been shown to be regulated by oxygen availability in *T. reesei *[16]. The *trx2 *gene encoding thioredoxin was chosen as a potential marker for oxidative stress based on *S. cereviciae *transcriptional data [18]. In batch cultures (Figure 3) and in the batch phase of fed-batch cultures the expression of these three genes increased 1.6 to 2-fold between 18 to 45 h, while the biomass increased from approximately 3 to 20 g l^-1 ^and pO_2 _decreased from 90 to 30 %. A 20–35% decrease in the expression level relative to polyA RNA was observed for these 3 genes after the maximal expression was measured (Figure 3A).

The maximal expression levels of these genes in batch cultures were compared to the corresponding levels measured in steady state of chemostat cultures with high and low cell density, and in anaerobic conditions (TRAC data of chemostat and anaerobic cultures for this comparison from Rautio *et al*. [16]) as well as in shake flask precultures (Figure 3B). In anaerobic conditions the expression levels of *hsp70 *and *hem6 *were 3.5 and 2.8 -fold higher than the maximal expression levels measured in batch or fed-batch cultures. Comparable *hsp70 *and *hem6 *expression levels were measured between high cell density (16–20 g l^-1^) batch or fed-batch cultures and chemostat cultures with 30% pO_2_, whereas at low cell density (4 g l^-1 ^and a pO_2 _of 80%) the expression level of *hsp70 *was 2.7-fold lower and that of *hem6 *was appr. 2-fold lower than in the high cell density aerated cultures. In the shake flask cultures (~2 g l^-1^) *hem6 *expression levels were comparable to those observed in high density cultures, whereas the *hsp70 *transcript level was 2-fold lower. This indicates that other stress factors besides oxygen limitation resulted in up-regulation of the *hsp70 *mRNA level in the high density cultures. *Trx2 *expression was similar in all aerobic bioreactor cultures, but was on average 1.5-fold higher in aerobic than in anaerobic or shake flask culture conditions.

### Comparison of secreted enzyme production rate and mRNA expression

**Figure 4:**
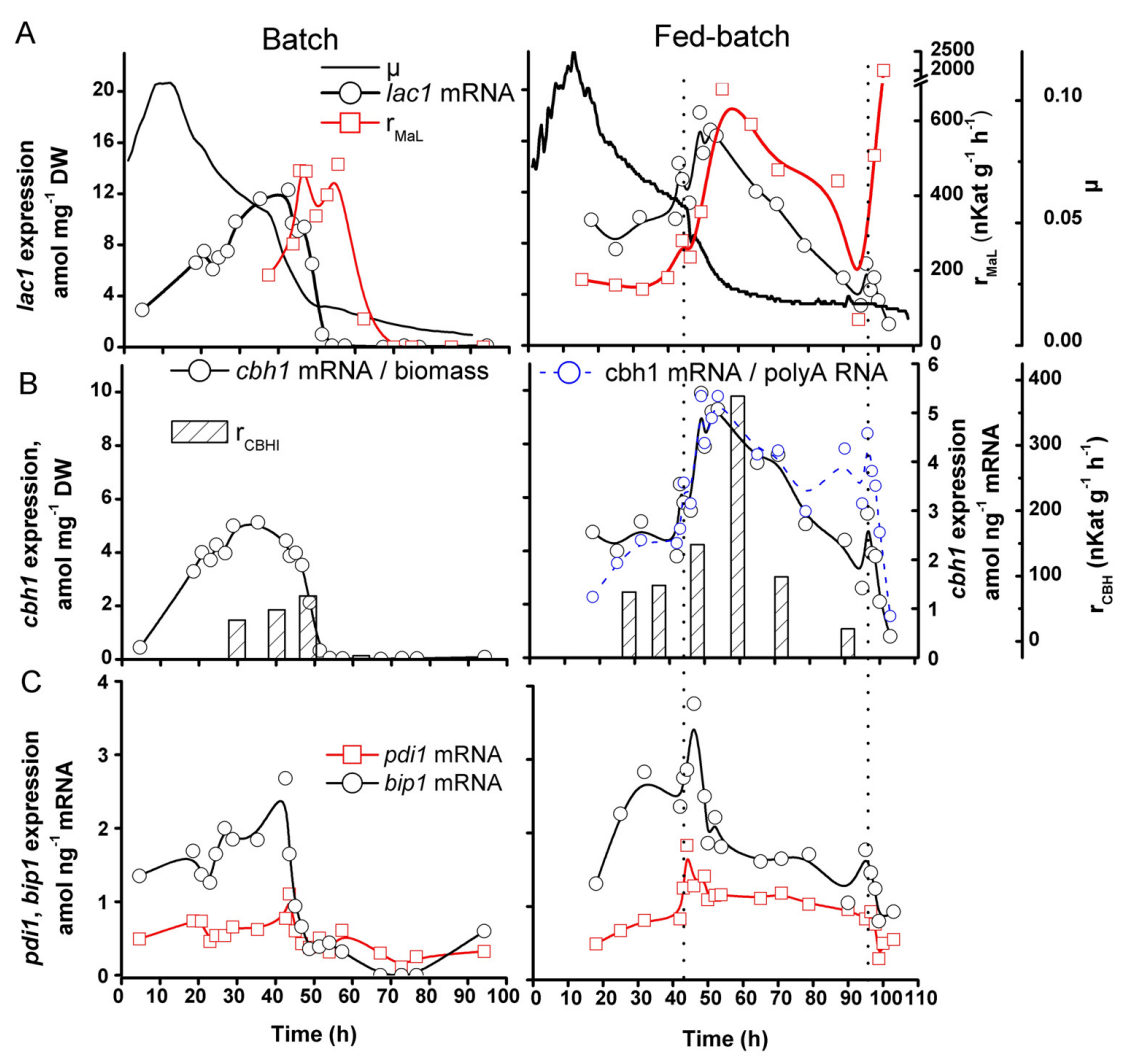
**Expression of the recombinant *M. albomyces *laccase gene *lac1*, the cellulase gene *cbh1 *and protein folding factor genes *pdi1 *and *bip1 *in batch and fed-batch cultures of *T. reesei***. A. *lac1 *mRNA level relative to biomass, specific extracellular production rate of laccase r_*MaL*_, specific growth rate μ. B. *cbh1 *mRNA relative to biomass (batch and fed-batch) and relative to polyA RNA (fed-batch) and specific extracellular production rate of CBHI. C. *pdi *and *bip1 *mRNA relative to polyA RNA. Dotted, vertical lines in the fed-batch culture represent the start and end of lactose feed.

The transformant strain LLK13/295 used in these studies produced *M. albomyces *laccase under the cellobiohydrolase (*cbh1*) promoter, as well as all the native cellulases, including cellobiohydrolase I (CBHI). *Cbh1 *promoter is induced by cellulose and by oligosaccharides and disaccharides derived from cellulose, such as cellubiose or sophorose. Also several other disaccharides such as lactose induce *cbh1 *expression [19]. Transcript levels of the genes (*cbh1*, *lac1*) expressing these secreted enzymes and two folding factors (protein disulfide isomerase *pdi1 *and protein chaperon *bip1*) were monitored during batch and fed-batch cultures and were compared with specific extracellular production rates of laccase and CBHI (Figure 4). Both biomass (*lac1, cbh1*) and polyA RNA (*bip1, pdi1, cbh1*) concentrations were used to normalise gene expression so that *lac1 *and *cbh1 *could be readily compared with biomass specific production rates and to allow physiological interpretation of the data.

Expression of *cbh1*, *lac1*, *pdi1*, and *bip1 *increased during the first 40 h of growth in batch cultures. In the fed-batch culture there was a 1.8 and 2.6 -fold increase in mRNAs for *lac1 *and *cbh1 *respectively, after the lactose feeding was started, whereas their expression decreased in the batch cultures. There were corresponding increase in the specific extracellular production rates of laccase (3.8 -fold) and CBHI (4.3 -fold), after starting of the lactose feed in the fed-batch culture. The highest amounts of *lac1*, *cbh1, pdi1 *and *bip1 *mRNAs were measured between 7 and 12 h after the start of the lactose feed. 36 h after the lactose feed had been started both the gene expression (mol/biomass) and the specific enzymes production rates (nkat h^-1 ^g^-1 ^DW) had decreased to levels similar to those observed during the batch phase.

To study the variation between the biomass and polyA mRNA based normalisation they were compared in the normalisation of the major cellulase *cbh1 *expression in the fed-batch culture (Figure 4B). An expected difference between the profiles of *cbh1 *to biomass and *cbh1 *to polyA RNA ratios was observed at the end of the culture. *Cbh1 *to biomass ratio showed a faster decreasing trend after about 70 h than *cbh1 *expression relative to polyA RNA, since increasing proportion of the biomass became metabolically inactive and polyA was produced only by the metabolically active part of the culture. It should be noted, however, that *e.g*. increase in a gene mRNA to polyA RNA ratio is not necessarily a sign of specific up-regulation, but can also indicate slower down-regulation than for the majority of genes. However increase or decrease in this ratio indicates the growing or decreasing physiological importance of the gene product in the particular conditions.

The specific production rate of both CBHI and laccase followed trends similar to the *cbh1 *and *lac1 *gene expression profiles, although a delay was observed between the gene expression and the corresponding extracellular enzyme production rate. For example the specific extracellular laccase production rate, which was more frequently measured than CBHI, was highest 6 to 10 h after the highest gene expression level was measured. An increase in laccase production rate was consistently observed at the end of the fed-batch cultures after lactose feed was stopped (Figure 4). This was not observed for CBHI.

### Monitoring responses caused by starvation

**Figure 5:**
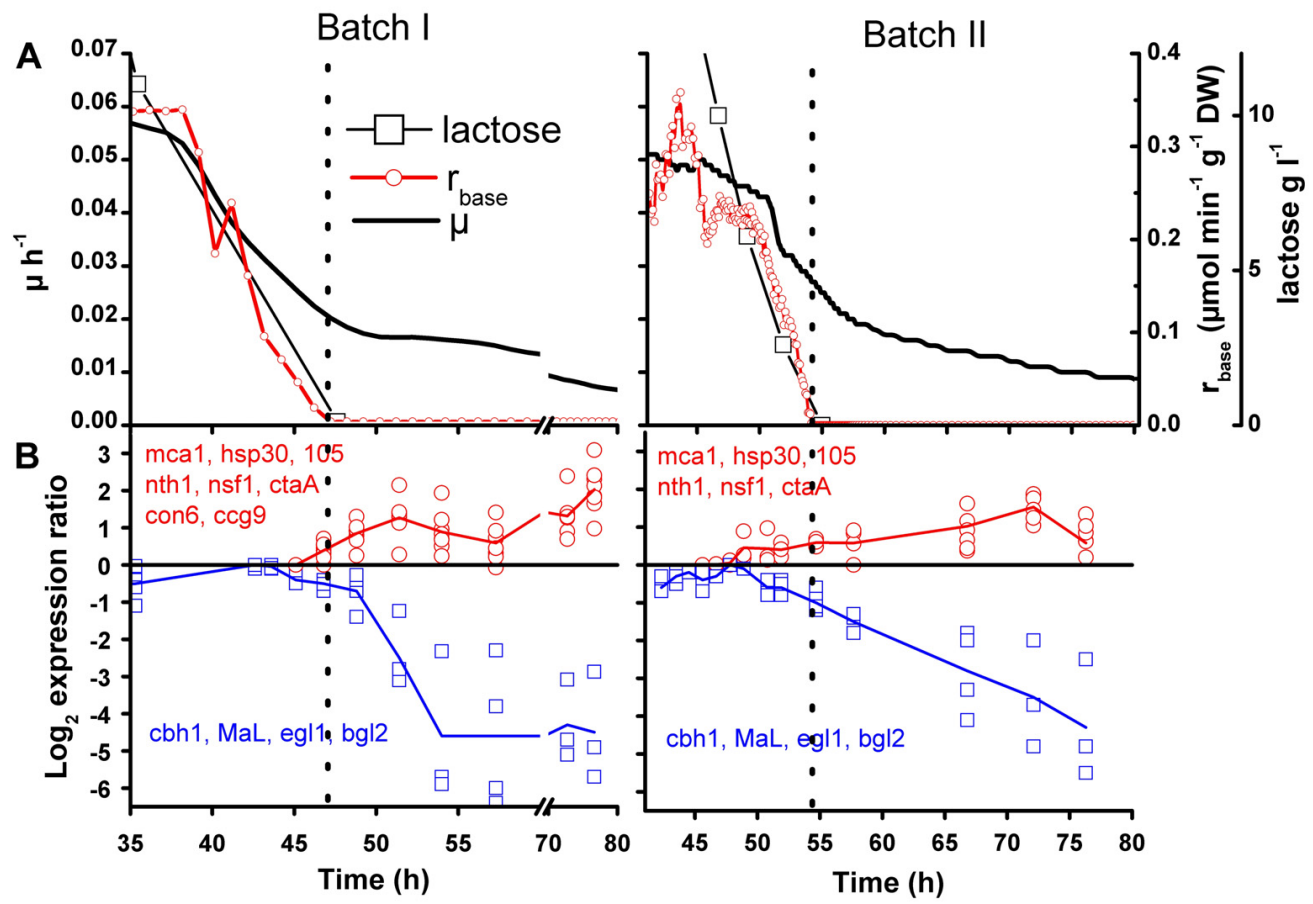
**Responses of marker genes to starvation in two batch cultures (I and II) of *T. reesei***. A. Specific growth rate μ, lactose concentrations, specific base consumption rate r_base_. B. Expression of cellulase and laccase genes as log_2 _ratio to maximal expression measured in the cultures (blue symbols). Expression of genes showing >2-fold increasing mRNA level relative to polyA RNA after exhaustion of lactose as log_2 _ratio to the expression level in a sample taken before the observed increase (red symbols).

The highest expression levels for genes coding the cellulases, laccase and folding factors were detected at growth rate 0.05 – 0.03 h^-1^, at low lactose concentration in batch and lactose limiting in fed-batch cultures (Figure 4). To determine the responses of the marker genes during the transition from deceleration to stationary phase, samples were taken frequently around the time when lactose was expected to be exhausted in batch cultures (Figure 5). Gene expression levels are presented relative to overall gene expression (polyA RNA), because the responses of metabolically active cells during starvation was of interest.

The time when the base consumption rate had decreased to zero and the lactose was undetectable was considered to be the time at which starvation began. The expression of genes coding for carbohydrate degrading enzymes was at this time on average 1.5 -fold lower than the maximal expression level measured during these cultures. The time of maximal expression of *cbh1*, *lac1*, *egl1 *(endoglucanase) and *bgl2 *(β-glucosidase) was 3.6 to 6.4 hours prior to the start of starvation, when μ was 0.03 – 0.045 h^-1^, the rate of base consumption was between 0.1 and 0.2 μmol min^-1 ^g^-1 ^DW and the lactose concentration was below 6 g l^-1^. Along with cellulases and laccases the majority of the genes analysed showed down-regulation during starvation, including the folding factors (*pdi1*, *bip1*), oxidative stress genes (thioredoxin reductase *trr1 *and thioredoxin protein *trx2*) and glycolysis genes (*eno1*, *gpd1*) (data not shown).

However, six to eight marker genes out of the 34, in addition to *acs1 *(Figure 2), showed more than 2 -fold increased mRNA levels during starvation (Figure 5). The trend of increasing expression of these genes started 0.5 to 5 hours before base consumption stopped. These genes coded for heat shock proteins *hsp105 *and *hsp30*, metacaspase (*mca1*) involved in apoptosis, neutral trehalase (*nth1*), membrane fusion factor (*nsf1*), copper transporter (*ctaA*) and two conidiation related proteins (*ccg9 *and *con6*, measured only in batch I).

### Monitoring of a continuous culture

**Figure 6:**
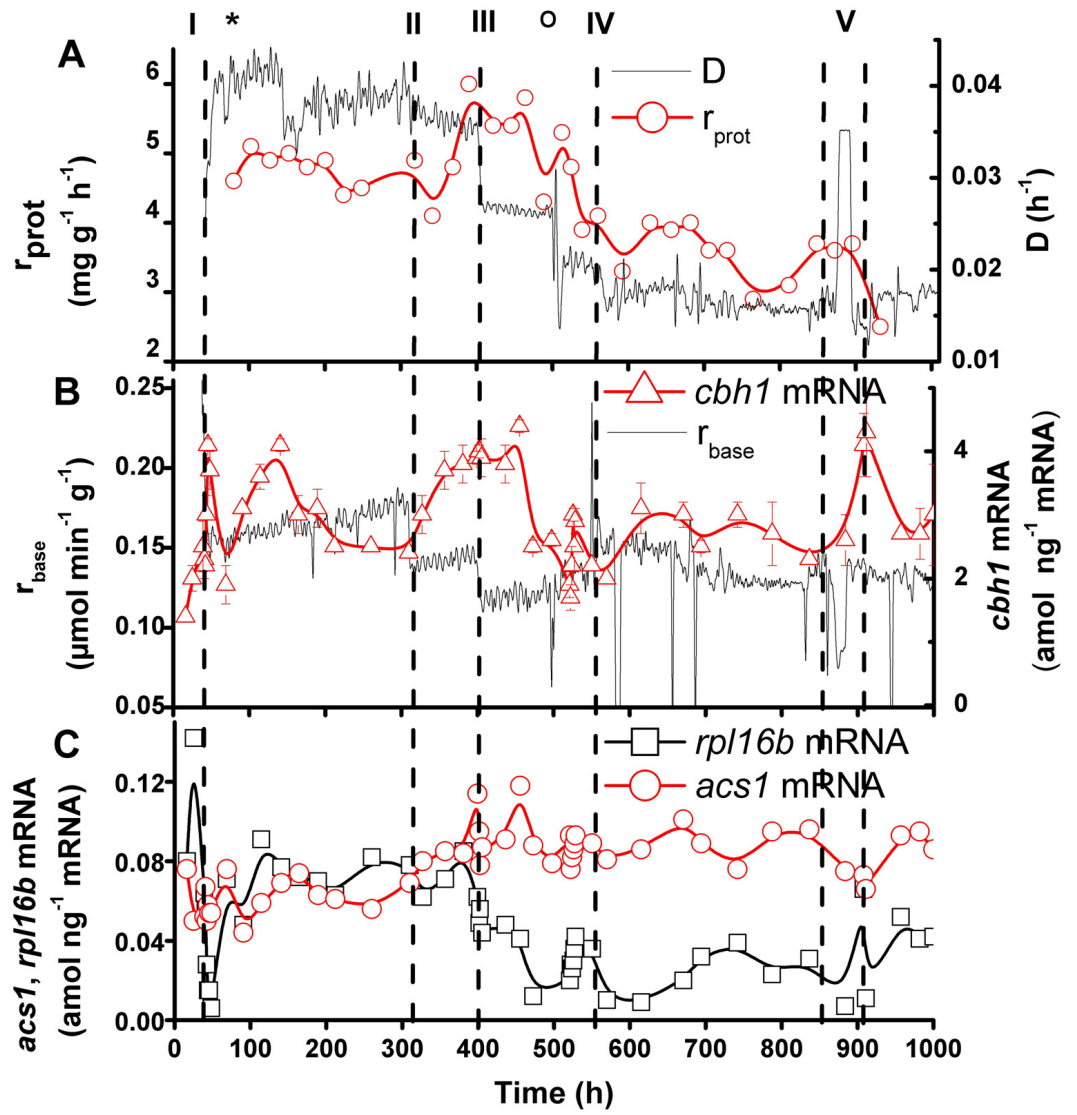
**Expression of *cbh1*, ribosomal protein mRNA and *acs1 *in a continuous culture of *T. reesei *run for 1000 h. **A. Culture dilution rate D, specific productivity of extracellular proteins r_prot_(mg h^-1 ^g^-1 ^DW). B. Specific base consumption rate r_base_(μmol min^-1 ^g^-1 ^DW), *cbh1 *mRNA expression relative to polyA RNA. C. *acs1 *and *rpl16b *mRNA expression relative to polyA RNA. I Start of continuous medium feed. II, III Changes of base consumption rate criterion (DELTABAS). IV Reduction in nitrogen provision. V Temperature gradient from 28°C to 24°C. Process disturbances (*) and (O).

Continuous culture was used to study further the effect of growth conditions on the productivity of extracellular proteins and to optimise their production. Different growth conditions were applied during the culture, *i.e*. altering the medium feed rate and composition and process temperature. In addition, unexpected disturbances occurred during the process, which was maintained for 1000 h. The TRAC assay was used to monitor expression 20 genes in a single pool, including markers that showed predictive value for cellulase productivity (*cbh1*), specific growth rate (*rpl16a*), and starvation (*acs1*) (Figure 6). A more detailed description of the process parameters in the continuous culture will be published elsewhere (M. Bailey, unpublished results).

The feed rate of the culture was controlled by the rate of base consumption (see Methods). The medium feed to the fermentor started at 41 h (Figure 6I) when the rate of base consumption decreased below the DELTABAS criterion value (0.02 ml (5% NH_4_OH) l^-1 ^min^-1^). This criterion kept the dilution rate (D) at approximately 0.04 h^-1 ^and the specific base consumption rate, r_base_, was 0.165 μmol min^-1 ^g^-1 ^DW. The criterion for base consumption was decreased in two steps: at 309 h by 25% (Figure 6II) and at 407 h by additional 20% (Figure 6III). The average r_base _value decreased to 0.14 and 0.12 μmol min^-1 ^g^-1 ^dry weight as a result of these two changes and the average dilution rate decreased to 0.036 and 0.026 h^-1^. The *cbh1 *mRNA level increased 1.6-fold (Figure 6B) and the specific extracellular protein production rate (r_prot_) increased 1.2 – 1.5 -fold (Figure 6A) after the first step (II). After the second reduction in base consumption rate (III) there was no immediate effect on *cbh1 *mRNA or extracellular protein production rate, but after 48–57 h (1.2–1.5 residence times) these values decreased to the level measured prior to the changes in the base consumption rate (before 309 h).

At 550 h the organic nitrogen concentration was halved in the medium (Figure 6IV), which decreased D to an average value of 0.017 while the r_base _increased to an average level of 0.134 μmol min^-1 ^g^-1 ^DW. The lower level of nitrogen in the medium will have been partly compensated for by the increased base (NH_4_OH) consumption. The specific production rate of extracellular proteins was unaffected by this change and the only marker gene which responded to the lowered N concentration was the amino acid permease *gap1*, the mRNA level of which was increased 1.8-fold 65 h after change in the medium (data not shown).

The expression profile of the ribosomal protein mRNA (*rpl16a*) followed the changes in the dilution (=growth) rate throughout the culture, consistent with earlier observations from batch cultures (Figure 1). The gene coding for acetyl-CoA-synthase (*acs1*), which showed its highest expression at a low lactose concentration (Figure 2), was somewhat induced when the dilution rate was reduced (Figure 6C). Two other genes (*hsp30*, *nsf1*) showing increased mRNA level under starvation conditions (Figure 5) were also transitorily induced after 309 h when the rate of medium feed decreased (data not shown).

Finally at 861 h the temperature of the culture was reduced from 28 to 24 °C over a 50 h period (Figure 6V). A number of marker genes responded by increasing their expression levels. Some of these genes (*cbh1*, *egl1, lac1 *and vacuolar protease *vpa1*) returned to the expression levels measured before the temperature change in less than 45 h, whereas others (*hsp30, nsf1, bgl2, gpd1*) remained at the higher expression level, even after the temperature was increased to 28°C again for the remainder of the culture. However, it can not be concluded that these responses were specifically related to the temperature change, since a temporary increase in the dilution rate occurred simultaneously because of a process control problem when growth and base consumption were reduced at the lower temperature (see Figure 6V).

Two technical disturbances occurred during the process. The first occurred at 68 h when the vessel weight controller malfunctioned and the culture volume decreased to about 8 litres. The volume was increased to 10 l by medium addition, which caused increased expression of *e.g. cbh1 *and ribosomal protein mRNA (*rpl16a*) genes. Both reached stable expression levels within 70–90 hours (2.8 – 3.8 residence times) of the disturbance (Figure 6*). At 504 h, oscillation in the feed control mechanism was observed. Samples were collected at 1 h interval after this disturbance and temporarily increased expression in many of the marker genes (e.g. *cbh1, rpl16b, pdi1, gpd1*) was observed (Figure 6O).

## Discussion

In this work we monitored the expression of 34 marker genes during changing conditions in batch, fed-batch and continuous fermentations of the filamentous fungus *T. reesei *using the transcriptional analysis method TRAC. In production processes the environmental conditions in the fermentor may be variable, causing corresponding variability in physiological parameters. This makes conventional gene expression data normalisation methods, such as the use of house-keeping genes, unreliable. In this study we related the expression of specific genes either to the amount of biomass or to the total polyA RNA content used in the hybridisation. Relating marker gene expression to biomass can be more useful in prediction of culture performance parameters such as specific growth and production rates, whereas normalisation to polyA RNA predicts the physiological responses in metabolically active cells more accurately.

Both polyA RNA to biomass and polyA RNA to total RNA ratios were shown to be growth phase dependent, and batch-to-batch variation in growth and protein production was also evident at the level of overall mRNA expression (Figure 1). Quantitative determination of total RNA per g biomass from mycelial biomass is not reliable, however comparing the polyA RNA per g biomass with polyA RNA per total RNA from the same culture (Figure 1) indicated that the total RNA to biomass ratio was not constant in changing environmental conditions of the batch cultures. It is thus beneficial to use a quantitatively measured factor to normalise expression levels between samples collected from different phases of a cultivation, rather than basing the normalisation on the assumption that a chosen factor is constant.

The normalisation based on polyA RNA is similar to the use of the expression levels of all genes for a normalisation in microarray experiments. However, for normalisation of genome-wide transcription data the distributions of the expression signal intensities between samples are equalised, *i.e*. it is assumed that global shifts in the mRNA population do not occur. This is a reasonable assumption *e.g*. in steady state conditions that can be achieved in continuous cultures, however disregarding global changes in mRNA levels during non-steady conditions (*e.g*. batch and fed-batch cultures) can result in the masking of true gene expression differences between compared samples [20]. On the other hand, variability in oligo dT-based mRNA capture can be caused by decreasing polyA tail length, that has been observed in stationary phase yeast cells [21]. However, since deadenylation leads to degradation of the mRNA body [22], and the polyA tail is required for translation initiation [23], the physiological relevance of mRNA species with short or no polyA tail is presumably insignificant, when the mRNA synthesis rate is low.

Expression of the native cellulase gene *cbh1 *and the recombinant laccase gene *lac1 *showed correlation with the specific extracellular production rates of the corresponding enzymes. There was a delay of 6 to 10 h between increased level of *lac1 *transcripts and laccase production (Figure 4). *M. albomyces *laccase is processed at its C-terminus and is presumably activated by the processing [24], and a delay in this processing might contribute to the difference between the *lac1 *expression and laccase production profiles. C-terminal processing of laccase could also explain the strong increase of its activity in the fed-batch cultures after termination of the substrate feed, which was not observed for CBHI (Figure 4). Gene expression of the secreted enzymes was highest when the growth rate was decreasing from 0.05 to 0.03 h^-1 ^in batch and fed-batch cultures (Figures 4 and 5) or was constant between 0.026 and 0.036 h^-1 ^in the continuous culture (Figure 6). This is in accordance with the optimal growth rate (0.031 h^-1^) for production of extracellular proteins observed in chemostat cultures of *T.reesei *[25]. When the growth rate was below 0.03 h^-1 ^decreased gene expression of cellulases and recombinant laccase was observed in addition to the responses associated with starvation (Figure 5). This data indicates that the "physiological window" in which the productivity of these extracellular proteins is optimal is narrow. In general the relation between specific product formation rate of a secreted protein and specific growth rate seems to be more dependent on the protein and its transcriptional promoter than on the organism. The specific product formation rate of a secreted protein can increase consistently with increasing growth rate, as shown *e.g*. for Fab fragment production in *Pichia pastoris *[26] and β-galactosidase production in *Escherichia coli *[27]. Whereas for other secreted proteins, such as α-galactosidase of *P. pastoris*, the specific product formation rate is at highest at low growth rates [28].

The DELTABAS feed control system, uses the rate of base consumption to try to maintain a culture in the optimal production phase by limiting the growth of the culture but avoiding total carbon source depletion [29]. During the optimal production phase the base (NH_4_OH) was consumed at a rate of 0.1 – 0.2 μmol min^-1 ^g^-1 ^DW (Figures 5 and 6). However, in the fed-batch and continuous cultures in which this feeding strategy was applied, the optimal production was maintained for rather short periods of time. In the continuous culture most of the time the growth (and base consumption) rates were either too high or too low (Figure 6). During the feeding phase in the fed-batch cultures the growth rate was mainly below 0.025 h^-1 ^(Figure 4), being too low for optimal production. Induced expression level of some of the starvation markers during the lactose (fed-batch, data not shown) or medium feed (*acs1*, continuous culture, Figure 6C) further indicated that too little substrate was available for the culture at this low dilution rate. However, at the end of the fed-batch cultures it was observed that the metabolically active part of the culture was still expressing cellulase genes (*cbh1*, Figure 4B) at a relatively high level, suggesting that improved productivity would be attained if culture viability could be maintained for a longer time.

Altogether 9 out of 34 marker genes (Figures 3 and 6) were observed to have more than 2-fold increased mRNA level under starvation and slow growth conditions, indicating their increasing importance under these conditions. Of the induced genes, *nth1 *codes for neutral trehalase, which is involved in degradation of storage carbohydrates [30]. *CtaA *codes for a copper transporter [31]. and in these fermentations the copper requirement may have been increased because of production of the copper-containing laccase [32]. The cysteine protease encoded by *mca1 *[33] is presumably involved in programmed cell death. In yeast the corresponding Mca1p is under post-translational regulation [33], but in *T. reesei *it would appear also to be subjected to transcriptional regulation. In *Neurospora crassa *the expression of the clock controlled gene *ccg9 *has been shown to be induced during glucose deprivation [34]. The increased level of *con6 *mRNA may be a response to nitrogen starvation, as shown by Bailey-Shrode and Ebbole [35]. High gene expression of *hsp30 S. cerevisiae *homologue (HSP30) has been observed in late stationary phase yeast cells [36] and *hsp105 *is homologous to the mammalian glucose-regulated protein GRP170, for which up-regulation has been shown when cellular UPD-glucose is reduced [37]. *S. cerevisiae *SEC18 (a homologue of *T. reesei nsf1*) is involved in starvation-induced degradation of tryptophan permease in yeast [38]. The *acs1 *gene has been shown to be strongly induced in glucose starvation in *T. reesei *[17]. The induction of these genes elucidates some of the physiological events during starvation and slow growth: mobilisation of storage carbohydrates, acquisition of nutrients from the medium, stress responses, onset of conidia formation and triggering of apoptosis. The consistent induction of these genes preceding the complete exhaustion of the carbon source can make them useful markers in optimisation of substrate feeding strategies, when their expression is monitored continuously.

The heme biosynthesis gene HEM13 of *S. cerevisiae *has been shown to be induced under anaerobic conditions [39] and the Ssa type Hsp70 proteins have been shown to be part of a complex that regulates transcription of aerobic genes [40]. The homologues of these genes are induced by anaerobic conditions in *T. reesei *(Figure 3). Based on the expression of these genes during the batch, fed-batch and continuous processes, 30% dissolved oxygen (DO) concentration even at relatively high cell density (DW ~ 20 g l^-1^) is apparently sufficient to maintain aerobic culture conditions, although at lower density (4 g l^-1^) and higher DO (80%) the expression of these genes was more than 2-fold lower. Strong induction of these anaerobic marker genes and decreased levels of the cellulase gene mRNAs were observed only when DO was lower than 10% (data not shown). In large scale industrial fermentations with thick filamentous slurry, inhomogeneity of the system can cause local limitations in oxygen supply [41]. Thus monitoring of the expression of these markers in such fermentations could be useful in devising intervention strategies which would maintain better aeration and productivity. Simultaneously with the increase in *hsp70 *and *hem6 *expression, moderately increased expression of the thioredoxin gene (*trx2*) was observed (Figure 3), which indicated a response to accumulation of reactive oxygen species presumably because of culture ageing [42].

The TRAC method was shown to be an efficient tool for monitoring sets of marker genes in different bioprocess conditions. The possibility to analyse simultaneously in 96-well format large number of samples, allows frequent sampling during the cultivations. Crude lysed cell material can be directly used in the hybridisations, thus RNA extraction and cDNA conversions are avoided. Detection of multiple mRNA targets from a single sample and low hands-on time of the protocol makes the assay cost-effective. For off-line monitoring, the advantages of the system are most clearly demonstrated in the analysis of the continuous culture. The protocol starting from the lysis of all the 48 mycelial samples to analysis of expression of 18 genes in duplicate (1728 expression levels) was carried out in 7 h with 1 to 2 h hands-on time. For at line monitoring (less than 10 samples at once) the entire protocol (from sampling to results) can be carried out in approximately 2 h. Increasing the degree of automation by combining some or all of the 4 steps (cell lysis, hybridisation, sample treatment, CE analysis) would make the overall protocol time even shorter and frequent at-line monitoring more practical. Such an analysis system would have a wide application potential.

We have demonstrated here that by marker genes analysis it is possible to evaluate various physiological factors of the culture, such as nutrient and oxygen limitation, growth and extracellular protein production rates in the changing environmental conditions. Transcriptional analysis can provide a more complete picture of the physiological state of an organism than can be achieved by the external parameters that are measured from production processes. In addition to increasing the knowledge of gene regulation, gene expression data collected at high frequency may suggest strategies for optimising process parameters, such as medium composition and feeding strategy. Accumulation of microarray data from *T. reesei *and other industrially relevant microorganisms grown under various conditions will further help in the selection process of marker genes, the expression of which has a predictive value in the evaluation of the physiological state and performance of the cultures.

## Conclusion

We have monitored by the TRAC method the expression of a set of 34 marker genes, involved in different process relevant pathways, in batch, fed-batch cultures and continuous cultures of a filamentous fungus *T. reesei *transformant strain producing *M. albomyces *laccase. Many of the marker gene expression levels measured at frequent intervals showed to have value in prediction of consecutive physiological effects and process performance. mRNA levels of genes coding for industrially relevant secreted cellulases and recombinant laccase followed the trends in the corresponding extracellular enzyme production rates and was at highest in narrow specific growth rate range of 0.03 – 0.05 h^-^^1^. The specific growth rate of the fungal cultures was possible to evaluate on the basis of ribosomal protein mRNA expression. Increasing expression of altogether nine starvation related genes preceded the complete exhaustion of carbon source at least by some hours, indicating their usefulness in prediction of insufficient substrate feed for optimal production. Deficiency in oxygen supply was manifested by increased level of two oxygen sensitive genes *hem6 *and *hsp70*. The TRAC method was shown to be an effective tool in focused transcriptional monitoring of biotechnical processes and the data produced by this method supported the usefulness of intensive gene expression analysis for process optimisation work.

## Methods

### Strain, medium and cultivation conditions

*Trichoderma reesei *Rut-C30 transformant pLLK13/295 producing *Melanocarbus albomyces *laccase [43] was used in these studies. The inoculum was cultivated 3 × 200 ml flasks at 28°C, 200 rpm, for 2 days, starting from a stock spore suspension maintained in 15% glycerol at -80°C. The buffered inoculum medium contained 20 g lactose l^-1 ^and other components described previously [44].

The fermentor medium contained (g l^-1^): lactose 40, peptone 4, yeast extract 1, KH_2_PO_4 _4, (NH_4_)_2_SO_4 _2.8, MgSO_4_×7H_2_O 0.6, CaCl_2 _× 2H_2_O 0.8, CuSO_4_×5H_2_O 0.025 and 2 ml 2 × trace element solution l^-1 ^(Mandels & Weber, 1969). pH was adjusted to 5.5 – 6 with NH_4_OH and H_2_PO_4 _and the cultivation temperature was 28°C. Dissolved oxygen level was maintained above 30% with agitation at 600 r.p.m., aeration at 0.5 vvm (volumes of air per volume of liquid per minute) and 0–20% O_2_-enrichment of incoming air. Foaming was controlled by automatic addition of Struktol J633 polyoleate antifoam agent (Schill & Seilacher, Germany, Hamburg) or polypropylene glycol (mixed molecular weights; [45]). Feeding of 24% (w/v) lactose solution into fed-batch fermentations and of complete culture medium in the continuous fermentation was controlled by the rate of base consumption in the culture using the algorithm described by Bailey and Tähtiharju [29]. The value of the DELTBAS variable [29] was calculated as the amount of base consumed per litre (kg) of medium or fermentor volume within 5 min intervals. Samples were taken frequently for dry weight, lactose, total protein, laccase, β-1,4-endoglucanase and cellobiohydrolyase I (CBHI) activity and gene expression measurements. Both batch and fed-batch cultures were performed as independent duplicates and the continuous culture was performed as a single culture maintained for 1000 h.

### Analyses of fermentations

Dry weights were measured by filtering and drying mycelium samples at 105°C to constant weight. Residual lactose in the culture filtrate was measured enzymatically (Lactose kit, Roche, Basel, Switzerland). Soluble protein concentration was measured using the Bio-RAD Protein assay (Hercules, CA). Laccase activity was calculated by measuring oxidation of 5 mM ABTS in 25 mM succinate buffer (pH 4.5) at 436 nm, using the absorption coefficient (ε) of 29 300 M^-1 ^cm^-1 ^[46]. Cellobiohydrolyase I (CBHI) activity was measured according to Bailey & Tähtiharju [29].

### Transcriptional analysis by TRAC

Biomass was harvested from fermentations for transcriptional analysis by anaerobically withdrawing medium containing 50–150 mg fresh biomass. Biomass was separated from medium by quick filtration with glass-fibre filter disks (Whatman GF/B 47 mm ∅, Kent, UK). The biomass was immediately washed with RNAse-free (dimethyl pyrocarbonite (DMPC)-treated) water, after which the biomass was transferred in tarred screw-cap tubes to liquid nitrogen and stored at -80°C. This sampling procedure took <5 min.

Transcriptional analysis was performed with the TRAC assay from crude cell lysates as described in Rautio *et al*. [12]. Frozen *T. reesei *mycelia were suspended (100 – 400 mg wet weight ml^-1^) in buffer containing 5 × SSC (750 mM sodium chloride, 75 mM sodium citrate), 2% (w/v) SDS and 66 U ml^-1 ^RNA guard RNase inhibitor (Amersham Biosciences, Buckinghamshire, UK). Mycelia were disrupted with a FastPrep cell homogenizer (6.5 m s^-1^, 45 s) (ThermoSavant, Dreiech, Germany) using 500 μl acid-washed glass beads (Sigma). 0.3 – 1 mg (wet weight) of lysed mycelium, containing 50 – 200 ng polyA RNA, was added to the hybridisation reaction with 4 pmol biotinylated oligo(dT) capture probe and 1 pmol of each 6-carboxy fluorescein (6-FAM) labeled detection probe. The hybridizations were carried out in 96-well PCR plates (ABgene, Epsom, UK) at 60°C for 30–40 min with shaking at 600 rpm (Thermomixer Comfort, Eppendorf, Hamburg, Germany) in 100 μl total volume, containing 5 × SSC, 0.2% (w/v) SDS, 1 × Denhardt solution (0.02% (w/v) Ficoll, 0.02% (w/v) polyvinyl pyrrolidone, 0.02% (w/v) BSA) and 3% (w/v) dextran sulfate.

The steps following hybridization, including affinity capture, washing and elution, were automated with a KingFisher 96 magnetic bead particle processor (Thermo Electron, Vantaa, Finland) in 96-well plates as follows: 1) affinity capture of hybridized RNA targets to 50 μg of streptavidin-coated MyOne DynaBeads (Dynal, Oslo, Norway) at room temperature (RT) for 30 min, 2) washing of the beads twice for 1.5 min in 150 μl of 1 × SSC, 0.1% (w/v) SDS at RT, 3) washing twice for 1.5 min in 150 μl of 0.5× SSC, 0.1% (w/v) SDS at RT, 4) washing once for 1.5 min in 150 μl of 0.1 × SSC, 0.1% (w/v) SDS at RT and 5) elution of probes to 10 μl of deionised formamide (Sigma) for 20 min at 37°C.

The eluents were analyzed by capillary electrophoresis with ABI PRISM 310 or 3100 Genetic Analyzer (Applied Biosystems, Foster City, CA). In order to compare individual samples and to calibrate the separation of the detection probes by size, GeneScan-120LIZ size standard (Applied Biosystems) was added to each sample. The identity of the probes was determined by the migration and the quantity by the peak area. In order to convert the measured peak area to the molar amount of a probe, the fluorescence signal intensity relative to molar amount was determined for each 6-FAM labelled probe.

Total polyA RNA quantification from prepared lysates was performed with the above TRAC protocol without addition of detection probes. The final elution of polyA RNA was performed in 50 μl DMPC treated water. RNA concentration in the eluent was quantified with a RiboGreen RNA quantitation kit (Molecular Probes, Leiden, the Netherlands).

The expression levels of the marker genes during the cultivations are presented here either as changes relative to a control sample or as molar amounts of the target specific probe detected. The expression levels presented as molar amounts can be compared between conditions for a given gene, but not between different genes, since the hybridisation efficiency between probe-target pairs can vary.

### Probe selection

The detection probe oligonucleotides, labelled at the 3' and 5' ends with 6-FAM, were synthesized by Thermo Electron (Ulm, Germany). The biotinylated Oligo(dT) capture probe was from Promega. The HPLC-purified oligonucleotide detection probes (Table 1) were mixed in three pools, according to their migration in capillary electrophoresis. Oligonucleotides were designed using the algorithms presented in Kivioja *et al*. [47,48]. The quality criteria used in probe selection were the following: melting temperature (T_m_) limits 60 – 70°C, GC% limits 38 – 62, maximum free energy change in hybridisation ΔG_H _[49] -15 kcal/mol and minimum target energy change A_c _[50] -10 kcal/mol. A maximum repeat size of 15 nt and a maximum similarity of 80% were used as probe specificity criteria. T_m _values were calculated with the nearest-neighbour method [49] using 10 nM nucleic acid and 750 mM salt concentrations.

## Authors' contributions

JJR carried out the TRAC analysis, all data analysis and drafted the manuscript. MB designed and carried out most of the bioreactor cultivations and growth and protein production analyses and helped to draft the manuscript. TK participated in the computer based design of the TRAC probes and helped to draft the manuscript. HS participated in the design of the TRAC analysis and helped to draft the manuscript. MP participated in the conception, design and coordination of the study. MS conceived of the study, and participated in its design and coordination and helped to draft the manuscript. All authors read and approved the final manuscript.
